# Site-specific deacylation by ABHD17a controls BK channel splice variant activity

**DOI:** 10.1074/jbc.RA120.015349

**Published:** 2021-01-13

**Authors:** Heather McClafferty, Hamish Runciman, Michael J. Shipston

**Affiliations:** Centre for Discovery Brain Sciences, Edinburgh Medical School: Biomedical Sciences, University of Edinburgh, Edinburgh, United Kingdom

**Keywords:** *S*-acylation, palmitoylation, acyl thioesterase, potassium channel, ion channel, Kcnma1, Kcnmb1, lipid modification, protein trafficking, post-translational modification (PTM), lipid, membrane trafficking, acyl protein thioesterase

## Abstract

*S*-Acylation, the reversible post-translational lipid modification of proteins, is an important mechanism to control the properties and function of ion channels and other polytopic transmembrane proteins. However, although increasing evidence reveals the role of diverse acyl protein transferases (zDHHC) in controlling ion channel *S*-acylation, the acyl protein thioesterases that control ion channel deacylation are very poorly defined. Here we show that ABHD17a (α/β-hydrolase domain-containing protein 17a) deacylates the stress-regulated exon domain of large conductance voltage- and calcium-activated potassium (BK) channels inhibiting channel activity independently of effects on channel surface expression. Importantly, ABHD17a deacylates BK channels in a site-specific manner because it has no effect on the *S*-acylated S0–S1 domain conserved in all BK channels that controls membrane trafficking and is deacylated by the acyl protein thioesterase Lypla1. Thus, distinct *S*-acylated domains in the same polytopic transmembrane protein can be regulated by different acyl protein thioesterases revealing mechanisms for generating both specificity and diversity for these important enzymes to control the properties and functions of ion channels.

Protein *S*-acylation, the dynamic and reversible post-translational modification of cysteines residues through addition of a fatty acid (typically the 16-carbon fatty acid palmitate) via a labile thioester bond, is an important modulator of the life cycle of many polytopic transmembrane proteins including receptors, transporters, and ion channels (1, 2, 3, 4). Indeed, a large repertoire of pore-forming and accessory ion channel subunits are known to be *S*-acylated controlling diverse channel properties and functions from assembly, through trafficking to the cell surface, to regulation by diverse signaling cascades. *S*-Acylation is mediated by a family of transmembrane zinc DHHC (Asp-His-His-Cys) acyl transferases (zDHHC) (5, 6, 7). For many transmembrane proteins we are beginning to understand the repertoire of zDHHCs that can show some selectivity for different ion channels and even *S*-acylated domains in the same protein. In contrast, there is a dearth of evidence for the acyl protein thioesterase enzymes that remove lipids from (deacylation) most polytopic transmembrane proteins including ion channels. Classically, two members of the serine hydrolase superfamily (LYPLA1 and LYPLA2) were thought to be the major acyl protein thioesterases that mediate deacylation of cytosolic cysteine residues in proteins, even though it was clear that many proteins were not controlled by these enzymes (8, 9, 10). Indeed, additional members of the broader serine hydrolase family, in particular the ABHD17 (α/β-hydrolase domain-containing protein 17) family, have been identified by activity profiling and shown to be functional acyl protein thioesterases that are targeted to the plasma membrane and deacylate a number of peripheral membrane and other proteins including Psd-95, N-Ras, Gap-43, and Map6 (11, 12, 13, 14). However, the regulation of polytopic transmembrane proteins, including ion channels, by α/β-hydrolase domain–containing protein or other acyl protein thioesterases of the serine hydrolase family and whether they display specificity are essentially unknown.

We have previously revealed that the trafficking, function, and regulation of the pore-forming subunit (Kcnma1) of the large conductance calcium- and voltage-activated potassium (BK) channel is controlled by *S*-acylation of two distinct domains (Fig. 1*A*). The intracellular loop between transmembrane domains S0 and S1 (S0–S1 loop) is *S*-acylated at a cluster of conserved cysteine residues that controls trafficking to the plasma membrane and functional assembly with regulatory β1-accessory subunits (15, 16). This domain is largely *S*-acylated by zDHHC23 that promotes membrane trafficking of the α-subunit alone and is a target for deacylation by Lypla1 resulting in channel retention in the trans-Golgi network (15). In contrast, the alternatively spliced stress-regulated exon (STREX) introduces an additional conserved tandem cysteine motif that is *S*-acylated by a number of zDHHCs, including zDHHC17, but not zDHHC23 (17, 18, 19). *S*-Acylation of the STREX domain enhances the apparent calcium sensitivity of the channel (17, 18, 19) and determines STREX variant channel regulation by AGC-family protein kinase–dependent phosphorylation (17, 20). However, acyl protein thioesterases that deacylate the STREX domain have not been identified.

**Figure 1:**
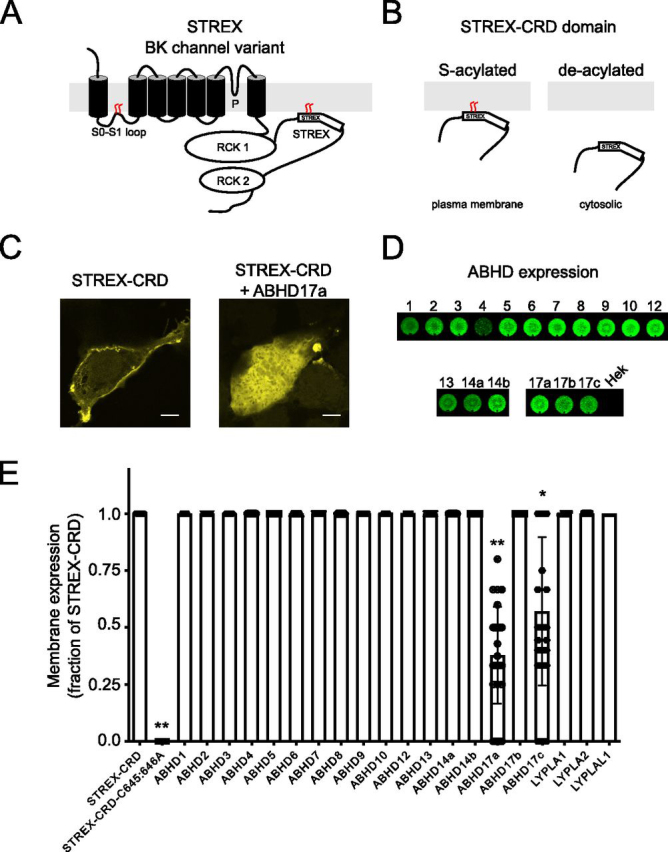
**ABHD17a controls plasma membrane association of BK channel STREX domain.***A*, schematic of pore-forming subunit of the STREX BK channel splice variant indicating the two *S*-acylated domains of the channel: the S0–S1 loop present in all channel variants and the alternatively spliced STREX insert in the intracellular C terminus of the channel located between the two regulator of potassium conductance (RCK) domains. *B*, the isolated *S*-acylated STREX–CRD fusion protein is associated with the plasma membrane when expressed in HEK293 cells and remains cytosolic when deacylated. *C*, representative confocal sections of HEK293 cells expressing the STREX–CRD (as a GFP fusion protein) and upon co-expression with ABHD17a. *Scale bars*, 2 µm. *D*, immunostaining of populations of HEK293 cells in 96 wells for overexpressed FLAG-tagged putative acyl thioesterases from the ABHD and Lypla families. *E*, quantification of membrane expression of STREX–CRD, as a GFP fusion protein, in HEK293 upon co-expression with ABHD and Lypla acyl thioesterase family members. The data are expressed as the fraction of cells expressing STREX–CRD at the plasma membrane compared with cells expressing STREX–CRD alone. The data are means ± S.D. (*n* = 23–54 independent experiments/group). **, *p* < 0.01; *, *p* < 0.05 compared with STREX–CRD alone using a nonparametric Kruskal–Wallis test with post hoc Dunn's test.

In this study, we sought to (i) characterize acyl protein thioesterases that deacylate the STREX domain; (ii) to establish whether these acyl transferases display specificity between the STREX and S0–S1 loop domains; and (iii) determine the functional impact of identified acyl protein thioesterases on channel trafficking and function. We demonstrate that ABHD17a is an acyl protein thioesterase that deacylates the STREX, but not S0–S1, domain of BK channels and in accordance with its specificity for the STREX domain controls channel activity rather than channel trafficking.

## Results

### ABHD17a deacylates the BK channel STREX domain

To define potential acyl thioesterases that control deacylation of the STREX domain of BK channels, we first employed an imaging-based screen that we have previously exploited to identify acyltransferases that *S*-acylate the STREX domain (17). In these assays we expressed the intracellular STREX–CRD domain of the BK channel (Fig. 1*B*) as a GFP fusion protein in HEK 293 cells. As previously reported, expression of the isolated STREX–CRD domain results in robust expression of the fusion protein at the plasma membrane of HEK293 cells (Fig. 1*C*) because of endogenous *S*-acylation of the fusion protein (17). Membrane expression of the fusion protein is completely abolished by site-directed mutagenesis of the tandem *S*-acylated cysteine residues (Cys^645^ and Cys^646^) to alanine in the STREX domain (STREX–CRD-C645A/C646A), resulting in predominantly cytosolic membrane expression.

To examine potential acyl thioesterases that deacylate the STREX domain we co-expressed the STREX–CRD fusion protein with a battery of candidate acyl thioesterases (Fig. 1, *D* and *E*), including members of the ABHD (11, 12) and classical LYPLA families that showed robust expression in our HEK293 cell assay system (Fig. 1*D*). Although activity-based profiling and functional assays have suggested that the ABHD17 family, as well as ABHD12 and ABHD13, may have thioesterase activity against *S*-acylated proteins for the vast majority of ABHD family proteins, their *S*-acyl thioesterase or other functions are not known (8, 11, 12). For example, ABHD 12 has also been shown to be a lysophopsholipid lipase, whereas the function of ABHD13 is not known. Significantly, of 19 putative acyl thioesterases tested, only ABHD17a and ABHD17c had a significant effect on reducing membrane expression of the STREX–CRD fusion as predicted for an acyl thioesterase that deacylates the STREX domain. ABHD17a and 17c have both been reported to control deacylation of a number of peripheral plasma membrane–associated proteins (11, 12). However, expression of ABHD17a resulted in a larger and more consistent reduction in membrane expression of the STREX–CRD fusion protein compared with ABHD17c (membrane expression expressed as a fraction (mean ± S.D.) of that for STREX–CRD alone was 0.38 ± 0.21 for ABHD17a (*n* = 28) and 0.57 ± 0.33 for ABHD17c (*n* = 24); *p* < 0.05 Kruskal–Wallis test with post hoc Dunn's test). In contrast, although ABHD17b was robustly expressed in HEK293 cells (Fig. 1*D*), this closely related ABHD family member had no significant effect on STREX–CRD membrane association. Moreover, Lypla1 and Lyplal1 that we have shown previously (15) can deacylate the S0–S1 loop of BK channels had no significant effect on STREX–CRD membrane localization.

To address whether ABHD17a and ABHD17c in fact deacylate the STREX domain of BK channels, we used acyl-RAC assays to address acylation of the full-length BK channel expressed in HEK293 cells in the presence and absence of ABHD17a and ABHD17c. In addition, we addressed whether ABHD17a or ABHD17c is specific for the STREX domain by assaying whether overexpression of these acyl thioesterases will also deacylate the S0–S1 loop domain of the BK channel to ask whether these ABHDs show specificity for distinct sites in the same polytopic transmembrane protein.

**Figure 2:**
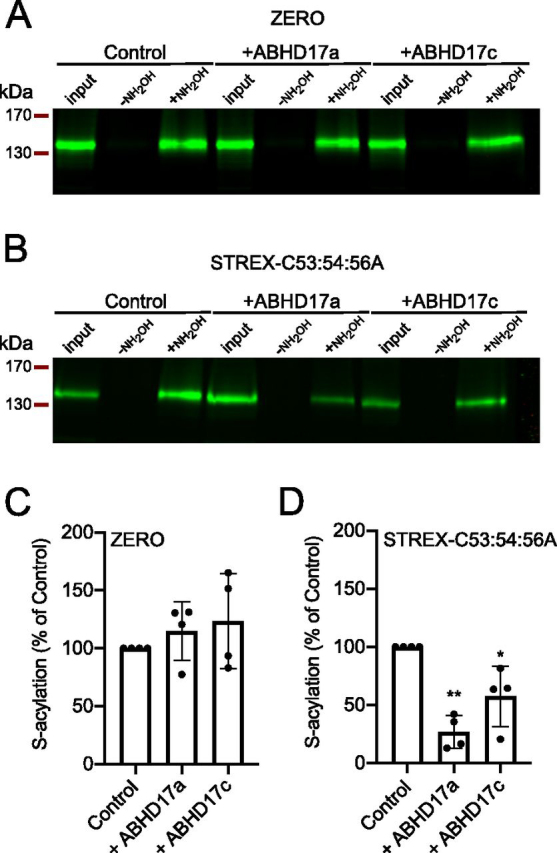
**ABHD17a deacylates the BK channel STREX, but not S0–S1 loop, domain.***A* and *B*, representative Western blots from an acyl-RAC assay of ZERO BK channel variant that lacks the STREX domain (*A*) or the STREX-C53A/C54A/C56A mutant that cannot be *S*-acylated in the S0–S1 loop (*B*), expressed in HEK293 cells alone or with ABHD17a or ABHD17c. Cell lysate input to the acyl-RAC assay is shown together with pulldowns following cleavage of thioester bond with hydroxylamine (+NH_2_OH) or in salt control (−NH_2_OH). *C* and *D*, quantification of *S*-acylation of ZERO (*C*) or STREX-C53A/C54A/C56A (*D*) in the presence of ABHD17a or ABHD17c. The data are expressed as percentages of *S*-acylation of corresponding ZERO or STREX-C53A/C54A/C56A channel alone (control) as means ± S.D. (*n* = 4 indepen-dent experiments/group). **, *p* < 0.01; *, *p* < 0.05 compared with STREX-C53A/C54A/C56A alone using nonparametric Kruskal–Wallis test with post hoc Dunn's test.

We first assayed whether the S0–S1 domain is deacylated by ABHD17a and 17c by expressing the ZERO variant of the BK channel. The ZERO variant is identical to the STREX variant except that it lacks the alternatively spliced STREX insert but is *S*-acylated at the conserved S0–S1 loop. In control cells, ZERO channels display robust and specific pulldown in acyl-RAC assays only when the thioester bond between the endogenous lipid and the cysteine residues in the S0–S1 loop has been cleaved by hydroxylamine (+NH_2_OH) (Fig. 2*A*). However, neither overexpression of ABHD17a or ABHD17c had any significant effect on ZERO channel pulldown by acyl-RAC assay (Fig. 2*C*), demonstrating that the S0–S1 loop is not a target for these ABHDs.

To address whether the STREX domain is specifically deacylated by ABHD17a or ABHD17c, we expressed STREX channels in which the *S*-acylation sites in the S0–S1 loop have been mutated to alanine (STREX-C53A/C54A/C56A construct). STREX-C53A/C54A/C56A channels are robustly *S*-acylated and thus pulled down in acyl-RAC assays from control HEK293 cells (Fig. 2*B*). Overexpression of ABHD17a and 17c both resulted in a significant (Fig. 2*B*) reduction in *S*-acylated STREX-C53A/C54A/C56A channels captured in these assays (*S*-acylation expressed as a percentage of STREX-C53A/C54A/C56A alone was 26.8 ± 14.1% for ABHD17a (*n* = 4, *p* < 0.01) and 57.5 ± 25.9% for ABHD17c (*n* = 4, *p* < 0.05; Kruskal–Wallis test with post hoc Dunn's test). This supports the data form our imaging screen that ABHD17a and 17c deacylate the STREX domain to control its association with the plasma membrane.

### ABHD17a or 17c do not control surface expression of STREX BK channels

The acyl-RAC data suggest that ABHD17a and 17c act specifically on the STREX domain of the BK channel and have no effect on the *S*-acylation of the S0–S1 loop of the BK channel. To further test this specificity, we first addressed whether ABHD17a or 17c controlled the surface expression of the BK channel. BK channel surface expression is attenuated in channels that are deacylated at the S0–S1 loop, as a result of increased retention in the trans-Golgi network (15). To assay BK channel surface expression, we exploited an on-cell Western approach to assay surface expression of epitope-tagged BK channels in cell population assays (16, 21). In these assays, the FLAG epitope at the extracellular N terminus of the BK channel is used to assay channels that are resident at the cell surface in intact cells, whereas the HA epitope at the intracellular C terminus of the channel allows assay of total BK channel expression in the same cells following cell permeabilization. The ratio of FLAG to HA signal thus provides a reporter of BK channel surface expression as a function of total channel expression.

**Figure 3:**
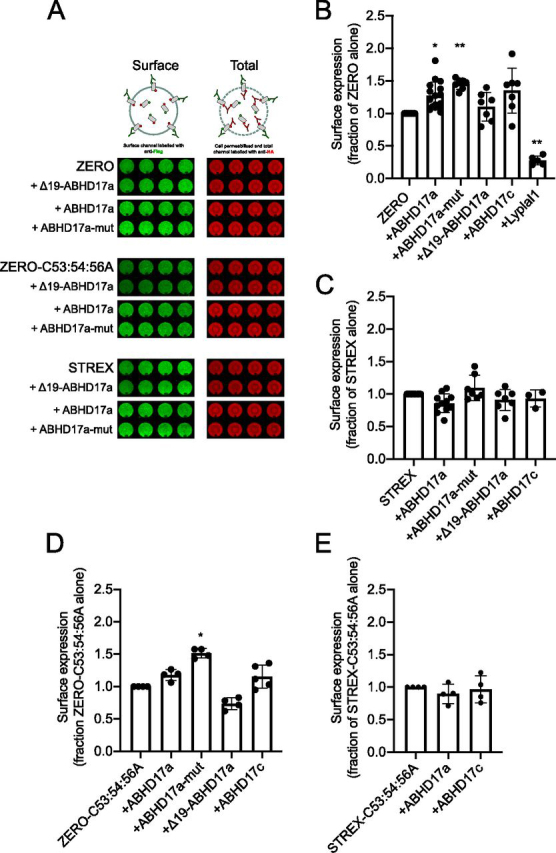
**ABHD17a–dependent deacylation does not control BK channel surface expression.***A*, representative on-cell Western assay of cell surface expression of epitope-tagged BK ZERO, ZERO-C53A/C54A/C56A, and STREX channels alone or co-expressed with WT β1 or co-expressed with WT ABHD17a, the catalytically inactive ABHD17a mutant (ABHD17a-mut) or the N-terminally truncated, targeting-deficient ABHD17a (Δ19-ABHD17a). Surface BK channel was quantified using an extracellular FLAG tag (*green*), whereas total BK expression was measured using an intracellular HA tag (*red*) following cell permeabilization. Four replicates from an individual experiment are shown. *B–E*, quantification of corresponding BK channel surface expression in the presence of acyl thioesterases and their related mutants as in *A* and expressed as a fraction of the corresponding BK channel subunit expressed alone. The data are means ± S.D. (*n* = 5–17 independent experiments/group). **, *p* < 0.01; *, *p* < 0.05 compared with corresponding BK channel group alone using nonparametric Kruskal–Wallis test with post hoc Dunn's test.

We first assayed cell surface expression of the ZERO variant of the BK channel that lacks the STREX domain (Fig. 3). In agreement with previous studies (15), co-expression with the cytosolic acyl thioesterase Lypla1 resulted in a significant reduction in ZERO channel surface expression as predicted as a result of deacylation of the S0–S1 loop (Fig. 3*B*). In the presence of Lypla1, ZERO channel surface expression was 0.28 ± 0.07-fold (*n* = 5) of ZERO channels alone. Surprisingly, overexpression of ABHD17a, but not ABHD17c, caused a small but significant increase in ZERO channel surface expression (Fig. 3, *A* and *B*). However, this increase in surface expression was also observed with the catalytically inactive mutant of ABHD17a in which Ser^170^ and Asp^255^ in the catalytic triad were mutated to alanine (ABHD17a-mut), suggesting the enhancement of surface expression is not dependent on deacylation *per se* (Fig. 3, *A* and *B*). In further support of this deacylation-independent effect, overexpression of ABHD17a-mut also had a small but significant enhancement of BK channel surface expression in ZERO channels that are *S*-acylation null (ZERO-C53A/C54A/C56A), ruling out the possibility that deacylation of the channel itself is involved in this stimulatory effect on surface expression (Fig. 3, *A* and *D*). ZERO or ZERO-C53A/C54A/C56A channel surface expression was not enhanced by the truncation mutant of ABHD17a (Δ19-ABHD17a) in which the N-terminal 19 amino acids, which include *S*-acylated cysteines important for membrane targeting (11), have been deleted (Fig. 3, *A*, *B*, and *D*). Taken together, this unexpected enhancement of ZERO channel surface expression likely results from either a chaperone effect on the channel or a potential dominant-negative effect of ABHD17a catalytic mutants on other components of the BK channel trafficking pathway.

In contrast, ABHD17a, its catalytic or membrane targeting mutants, and ABHD17c had no effect on STREX or STREX-C53A/C54A/C56A channel membrane expression (Fig. 3, *A*, *C*, and *E*). Taken together, these data demonstrate that ABHD17a and 17c do not inhibit BK channel surface expression through deacylation of the S0–S1 site in accordance with the lack of effect of these enzymes on S0–S1 loop *S*-acylation in acyl-RAC assays.

**Figure 4:**
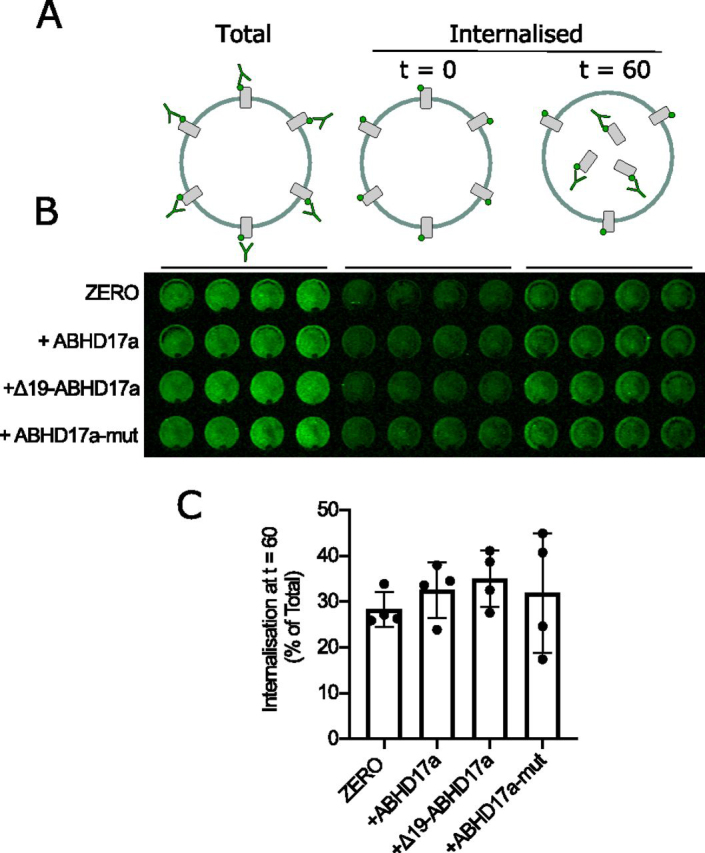
**ABHD17a does not control BK channel internalization.***A*, schematic of internalization assay. *B*, representative images from an internalization assay of epitope-tagged ZERO BK channels alone or co-expressed with ABHD17a and its mutants in HEK293 cells. Internalized BK α-subunit was quantified, after 60 min at 37 °C, using the extracellular FLAG tag (*green*) following acid-strip of surface staining in nonpermeabilized cells and normalized to surface expression at time 0. Four replicates from an individual experiment are shown. *C*, quantification of BK channel α-subunit internalization as a percentage of initial total surface expression. The data are means ± S.D. (*n* = 4 independent experiments/group).

To test whether the unexpected ABHD17a deacylation-independent enhancement of ZERO channel surface expression was a result of reduced ZERO channel internalization, we exploited a population-based assay of BK channel internalization in HEK293 cells (16). ZERO BK channels at the cell surface were labeled at their extracellular N-terminal FLAG tag epitope and allowed to internalize for 60 min at 37 °C before stripping of cell surface label allowing quantification of ZERO channel internalization (Fig. 4*A*). Approximately 30% of surface-expressed ZERO BK channels internalized over this 60-min assay in control HEK293 cells (Fig. 4, *B* and *C*). Overexpression of ABHD17a or its catalytic (ABHD17a-mut) or membrane targeting (Δ19-ABHD17a) mutant had no effect on ZERO channel internalization (Fig. 4*C*). This indicates that the enhancement of ZERO channel surface expression is likely due to deacylation-independent effects of ABHD17a on forward trafficking and/or delivery of the channel to the plasma membrane rather than through decreased internalization or stability at the plasma membrane.

### Deacylation of STREX domain by ABHD17a controls channel activity at the plasma membrane

**Figure 5:**
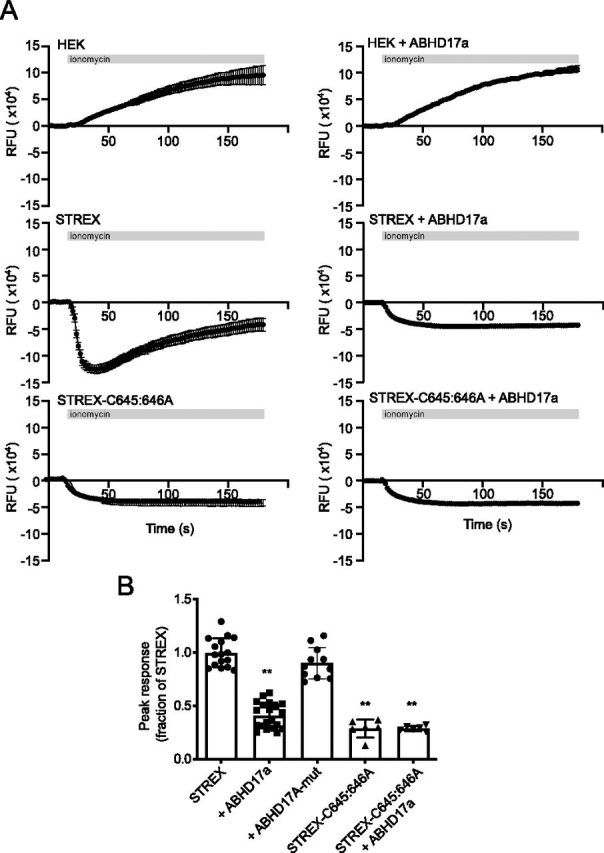
**ABHD17a-mediated deacylation of the STREX domain inhibits STREX channel function.***A*, representative experimental time courses of ionomycin (1 μm started at 16 s, *horizontal gray bar*)–induced changes in membrane potential of HEK293 cells transfected with STREX channel variants and ABHD17a using a 96-well format cell population Flexstation membrane potential assy. Positive changes in RFU denote membrane depolarization, whereas negative changes in RFU reflect membrane hyperpolarization. The assays were performed in physiological ion gradients with 2 mm extracellular calcium. The data are means ± S.D. from a single typical independent experiment with eight experimental replicates. *B*, quantification of peak hyperpolarization response to ionomycin in membrane potential assays expressed as a fraction of the peak hyperpolarization in cells expressing STREX channels alone. The data are means ± S.D. (*n* = 5–20/group). **, *p* < 0.01 compared with STREX using nonparametric Kruskal–Wallis test with post hoc Dunn's test.

Because ABHD17a had no effect on STREX channel surface expression, we asked whether ABHD17a-mediated deacylation of STREX has an effect on BK channel function and activity. We have previously shown that channels that cannot be *S*-acylated in the STREX domain are less sensitive to physiological voltages and intracellular free calcium levels compared with WT STREX channels (17, 19). To assay the effect of ABHD17a on STREX channel function, we used a membrane potential assay in HEK293 cells in which heterologously expressed BK channels are stimulated upon calcium entry mediated through the calcium ionophore ionomycin (22, 23). In this assay simulation of mock-transfected cells with ionomycin results in depolarization of the membrane potential (Fig. 5*A*) over the 180-s time course of the assay as a result of calcium influx. Overexpression of ABHD17a had no significant effect on the time course or magnitude of the ionomycin-induce depolarization of mock-transfected HEK293 cells (Fig. 5*A*). In cells overexpressing STREX channels, ionomycin results in a rapid and transient hyperpolarization of the membrane potential that typically remains significantly hyperpolarized compared with mock-transfected cells over the time course of the assay (Fig. 5*A*). Co-expression of ABHD17a with STREX channels resulted in a significantly attenuated peak hyperpolarization in response to ionomycin (Fig. 5, *A* and *B*) that is ∼60% smaller than the peak hyperpolarization observed with STREX channels expressed alone. In the presence of STREX and ABHD17a, the peak HEK293 hyperpolarization was 0.41 ± 0.12-fold, *n* = 20 (**, *p* < 0.01 Kruskal–Wallis test with post hoc Dunn's test compared with HEK293 cells expressing STREX channels alone). Importantly, the inhibitory effect of ABHD17a on STREX channel activity was completely abolished using the catalytically inactive ABHD17a mutant (ABHD17a-mut) (Fig. 5*B*). Moreover, the peak hyperpolarization observed on co-expression of STREX channels with ABHD17a was similar to that observed in STREX channel *S*-acylation null mutants that cannot be *S*-acylated in the STREX domain (STREX-C645A/C646A). Furthermore, ABHD17a had no significant effect on the hyperpolarization induced by ionomycin in STREX-C645A/C646A–expressing HEK293 cells, confirming that the inhibitory effect is mediated via deacylation of the STREX domain. Taken together, these data reveal that ABHD17a specifically deacylates the STREX domain of BK channels, resulting in functional inhibition of STREX channel activity.

## Discussion

**Figure 6:**
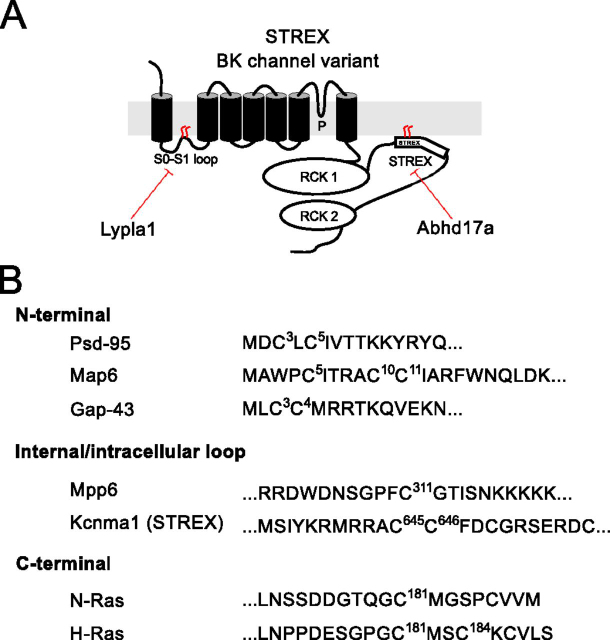
**Distinct acyl protein thioesterases control deacylation at distinct sites in BK channels.***A*, schematic of the STREX variant of the BK channel pore-forming subunit (Kcnma1) and sites of *S*-acylation. The STREX domain is deacylated by Abhd17a and the S0–S1 domain by Lypla1. *B*, amino acid sequence of other targets for ABHD17 acyl thioesterases showing the 10 most vicinal residues surrounding *S*-acylated cysteines. Mouse sequences and numbering are used throughout and include proteins with *S*-acylation at the (i) N terminus including Psd-95 (11, 12), Gap-43 (12), and Map6 (13); (ii) internal/intracellular loop cysteines such as Mpp6 (14) and the STREX variant of Kcnma1 shown here; and (iii) cysteines toward the C terminus of proteins including H-Ras and N-Ras (11, 12).

Using a combination of an imaging-based screen, acyl-RAC, and functional assays of BK channel trafficking and activity, we reveal that ABHD17a is a major acyl protein thioesterase that site-specifically deacylates the STREX domain of the BK channel to control channel activity. Together with previous work (15), these data reveal that in the same polytopic transmembrane protein different acyl protein thioesterases can control distinct sites with diverse functional impacts on channel behavior. In the case of BK channels, ABHD17a controls STREX domain and channel activity, whereas Lypla1 (and a splice variant of Lyplal1) controls the S0–S1 domain and channel trafficking/functional coupling to β-subunits (15, 16) (Fig. 6*A*). Strikingly, these domains are also regulated by distinct acyl protein transferases supporting an increasing body of evidence that both acyltransferases and acyl protein thioesterases can display some target specificity, as well as more promiscuous behavior.

To date, the majority of ABHD17 family targets for deacylation include a number of peripheral membrane proteins that are targeted to plasma membranes via *S*-acylated domains such as Psd-95, N-Ras, and Mpp6 (11, 12, 14) rather than transmembrane proteins (Fig. 6*B*). However, although ABHD17 members have been shown to display some selectivity between different proteins, the mechanism(s) by which such selectivity may occur is not known. Moreover, how ABHD17a and Lypla1, respectively, may discriminate between the S0–S1 and STREX domains in BK channels remains unclear. Several potential mechanisms may be involved. First, although some reports have suggested that Lypla1 can target the plasma membrane, the majority of studies suggest it is a largely cytosolic protein, whereas ABHD17a-c have a highly *S*-acylated N terminus and are shown to target in plasma membranes in a variety of cell types (9, 10). Thus, differential local accessibility of distinct BK channel loop domains from a cytosolic *versus* peripheral membrane location or differential association of Lypla1 and ABHD17a with these distinct BK channel domains may provide a mode of discrimination in cells.

Second, ABHD targets defined to date (Fig. 6*B*) have candidate target cysteine residues in either the N- or C-terminal tails of peripheral membrane proteins; there is no clear amino acid sequence consensus that might suggest discrimination of ABHD17 targets from other acyl thioesterases. Indeed, the classical acyl thioesterases Lypla1 and Lypla2 show both distinct and overlapping targets in accordance with their structural and sequence conservation with very similar surface polarity and substrate-binding regions (24). Lypla2 is likely more flexible than Lypla1 and because both have a single acyl-binding pocket may explain preference of both enzymes for monoacylated substrates, but both have reduced activity. However, recent studies using short fluorogenic peptides in assay screens has provided some insight into vicinal amino acids surrounding a single cysteine amino acid that may change the efficiency of hydrolysis between Lypla1 and Lypla2. For example, positively charged residues in the downstream P1′ and P2′ positions enhance hydrolysis, whereas bulky aromatic residues in the upstream P1 and P2 positions limit hydrolysis (25). Previously identified ABHD family targets (Fig. 6*B*) have residues vicinal to *S*-acylated cysteines that would not, in general, be highly favorable for hydrolysis by Lypla1 or Lypla2. However, the extent to which similar rules might apply to ABHD17 remains to be determined. Intriguingly, in our assays ABHD17b had no effect on the STREX domain in our imaging screens, whereas it is the major enzyme controlling PSD-95 deacylation (12). Thus, the potential differences in deacylation efficiency between ABHD17 isoforms remain to be explored.

Furthermore, whether ABHD or Lypla family acyl protein thioesterases have differential effects when target cysteines are in clusters of dicysteine motifs remains to be examined. Again, available sequences of ABHD17 target proteins (Fig. 6*B*) reveal that proteins with both clusters or dicysteine motifs are targets, such as in the STREX domain or in targets with lone cysteine residues. Moreover, the efficiency with which acyl thioesterases can catalyze hydrolysis of bound lipid species (depending on carbon backbone length or saturation) may also play an important role because in most cases the endogenous lipid, although often assumed to be palmitate, is not known for most *S*-acylated sites in proteins.

The ability of different acyl protein thioesterases to target distinct *S*-acylated domains in the same polytopic transmembrane protein suggests that deacylation of these two domains may be both spatially and temporally controlled as has been proposed for peripheral membrane proteins such as N-Ras and Psd-95, as well as the microtubule binding protein MAP6 (12, 13). The extent to which deacylation of these domains in BK channels is “constitutive,” dependent on the relative activity of competing zDHHC and acyl thioesterases or maybe dynamically controlled, for example, by environmental and/or signaling induced modulation of acyl protein thioesterase location and/or activity, remains to be explored (26).

In the course of these studies, we observed a consistent and significant effect of catalytically inactive (ABHD17a-mut) but not inappropriately targeted Δ19-ABHD17a to enhance the surface expression of the ZERO variant of BK channels. This small stimulatory effect on surface levels was not observed in the BK channel STREX, suggesting that overexpression of the catalytically null ABHD17a has distinct effects on the trafficking pathways controlling ZERO and STREX variant surface expression. Although *S*-acylation of the S0–S1 domain has been reported to modify lateral mobility and surface expression of BK channels (27, 28), ABHD17a-mut had no effect on ZERO channel internalization. Furthermore, this small enhanced surface expression upon ABHD17a-mut overexpression was also observed in *S*-acylation–deficient ZERO BK channels (C53A/C54A/C56A), demonstrating that this is independent of ZERO channel *S*-acylation but may result from changes in forward trafficking. Whether overexpression of ABHD17a-mut results in effects on other components of the BK channel trafficking pathway may act as a potential chaperone or may result from other activities or binding partners of ABHD17a warrants further investigation. For example, both Lypla1 and Lypla2, along with other ABHD family members display diverse functions, including hydrolysis of other lipids (8) but likely are functional monomers (24). Irrespective of mechanism, it does suggest that differences in forward trafficking for ZERO and STREX channels may exist.

In summary, we have identified ABHD17a as a major acyl protein thioesterase that deacylates the STREX domain of BK channels to control channel activity independently of changes in cell surface expression. Because ABHD17a and Lypla1 target different domains of the BK channel this may: (i) provide a suitable model to understand the rules controlling efficiency of acyl protein thioesterase to hydrolyze thioester lipid linkages in membrane proteins and (ii) allow us to unmask the mechanisms by which deacylation of distinct BK channels domains may control BK channel physiology and function.

## Experimental procedures

### Reagents

The general biochemical reagents used throughout this study were obtained from Sigma–Aldrich and were of analytical-grade quality unless stated otherwise.

### HEK 293 cell culture and transfection

HEK 293 cells were originally obtained from ATCC and cultured and transfected essentially as previously described (15, 16, 17). The cells were maintained in DMEM containing 10% fetal bovine serum (both Life Technologies), incubated at 37 °C in 5% CO_2_, and used between passage 18 and 30. HEK293 cells used in this study do not express endogenous BK channel subunits as determined by mRNA, protein, or functional assays (15, 16, 17). For transfection, the cells were typically plated on 6-well plates for 24 h before transfection with corresponding plasmid cDNA using PolyJet (tebu-bio). In all co-transfection assays, channel cDNAs were transfected at a 1:1 ratio with acyl thioesterases or empty pcDNA3.1 plasmid in controls.

### BK channel and acyl thioesterase constructs

The generation of BK channel ZERO and STREX variant subunits with an extracellular N-terminal FLAG- and intracellular C-terminal HA epitope tags in pcDNA3, together with *S*-acylation–deficient site-directed mutants and the STREX–CRD domain as a YFP fusion have been described previously (15, 16, 17). Original ABHD family clones as FLAG-tagged constructs in the pCAGGS vector were a generous gift of Prof. Masaki Fukata (12) and used in the initial imaging screens in Fig. 1. Lypla1, Lypla2, and Lyplal1 clones were as previously described (15). A catalytically inactive version of ABHD17a was made by mutating amino acids Ser^170^ and Asp^255^ in the catalytic triad to alanine by synthesizing the entire coding sequence (Twist Bioscience) containing the mutated amino acid codons and incorporating the FLAG tag and restriction sites EcoRI and NotI flanking the coding sequence and subcloned into pcDNA3.1. The targeting-deficient ABHD17a mutant (11, 12) that lacks the first 19 amino acids at the N terminus including *S*-acylated cysteine residues required for membrane targeting (Δ19-ABHD17a) was generated by PCR using a forward primer containing an EcoRI restriction site and a start methionine, 5′-AAAGAATTCACCATGGGCCGCATCGCGCGGCCAAG-3′, and a reverse primer to amplify a KpnI site in the original sequence, 5′-CCACTGCAGCACACTCATAACG-3′, were used to PCR-amplify the clone, subcloning back into an empty pCAGGS vector. For assays in which ABHD17a enzymes were co-expressed with FLAG and HA epitope-tagged BK channels, the FLAG tags of the corresponding full-length ABHD17a clones was engineered to a Myc epitope tag by PCR. Briefly, full-length constructs were amplified by PCR using the following primers: forward (including a BamHI site), 5′-GCCGGATCCGGCCACCATGAACGGC-CTGTCGGTGAGCGAGCTC-3′; and reverse, (including a XbaI site), 5′-TGGTCTAGATTA*CAGATCCTCTTCTGAGATGAGTTTTTGTTC*GGTGCGTTGG-3′ and the amplicons subcloned into pcDNA3.1. To generate Myc-tagged Δ19-ABHD17a, the forward primer used to generate Δ19-ABHD17a was used with the Myc reverse primer and cloned into pcDNA3.1 by restriction digest using EcoRI and XbaI. All clones were validated by DNA sequencing.

### STREX–CRD imaging assay

The cells were processed, imaged, and quantified essentially as described (17). In brief, HEK293 cells were plated on glass coverslips, transfected as above, and 48 h later fixed with 4% ice-cold paraformaldehyde for 15 min at room temperature. After quenching with 50 mm NH_4_Cl, the cells were either first probed for the epitope tag of the corresponding acyl thioesterase before mounting on microscope slides using Mowiol or Fluorsave. The cells were initially screened using an epifluorescence microscope with a 100× oil objective lens. Confocal images were acquired on either a Nikon A1 or Zeiss LSM510 laser scanning microscope, using a 63× oil Plan Apochromat (N.A. = 1.4) objective lens. The majority (>90%) of experiments were performed blind by an observer independent from the experimentalist undertaking the cell transfections. Membrane expression was typically observed in >95% of HEK293 cells transfected with the STREX–CRD fusion protein. To determine membrane expression upon co-expression with putative acyl thioesterases line scans of fluorescent intensity through four independent areas of the plasma membrane, the cytosol and nucleus of cells were analyzed using Fiji software (National Institutes of Health). To determine plasma membrane localization, a signal intensity at the cell periphery that was 2 S.D. greater than that in the cytosol in any of the four planes was defined as a cell with membrane expression. For each coverslip three to five random fields of view were analyzed to determine the number of transfected cells with plasma membrane localization of the STREX–CRD fluorescent fusion protein. The average percentage of transfected cells from each coverslip (minimum of 50 cells/coverslip) that displayed membrane expression was then normalized to the corresponding WT STREX–CRD control in each independent experiment.

### On cell Western cell surface expression assays

The assays were carried out as described previously (16, 21). Transfected HEK293 cells were plated into a 96-well poly-d-lysine–coated, black-walled, clear-bottomed plate (Greiner) and assayed 48 h post-transfection. The cells were stained on ice in growth medium (GM: DMEM + 10% fetal bovine serum) for the FLAG epitope applying mouse anti-FLAG-M2 (Sigma, 1:100 in GM), then washed in GM, and stained for 1 h with anti-mouse IRDye800CW goat IgG (Licor, 1:100 in GM). The cells were then fixed, permeabilized and blocked in Odyssey blocking buffer (OBB) before staining at room temperature for 1 h with anti-HA antibody (Immune Systems, 1:1000 in OBB) followed by anti-rabbit secondary IRDye690RD (Licor, 1:500 in OBB). Separate wells were stained with TO-PRO^TM^-3-iodide (1:500) to account for cell number. Cell staining was imaged on an Odyssey IR imager and were analyzed using Image Studio Lite version 5.2 (freeware from Licor). Staining intensity was normalized to cell number and background-subtracted before calculating the ratio of surface FLAG:HA total protein staining.

### BK channel internalization assay

The assays were carried out as described previously (16). Transfected HEK293 cells were plated into a 96-well poly-d-lysine–coated, black-walled, clear-bottomed plate (Greiner) and assayed 48 h post-transfection. All of the cells were stained on ice for the FLAG epitope, anti-FLAG-M2 (1:100 in DMEM + 10% fetal bovine serum (GM)), washed in GM, and then stained with anti-mouse IRDye800CW (1:1000 in GM). Ice-cold stripping buffer (0.1 m glycine, 0.1 m NaCl, pH 2.5, in PBS) was immediately applied to a subset of wells and then washed in growth medium to determine background staining. To allow endocytosis and internalization of labeled channels to occur, the cells were incubated at 37 °C in a humid chamber for 1 h. The cells were then cooled on ice, and a subset of cells was stripped to measure internalized staining, with a further subset remaining unstripped to measure total FLAG staining. All of the cells were then stained for 30 min on ice with NucRed^TM^ Live 647 ReadyProbes^TM^ reagent (ThermoFisher) to account for cell number. The cells were imaged on an Odyssey IR Imager and analyzed using Image Studio Lite version 5.2. For each well, staining intensity of FLAG was normalized to the NucRed signal. Background signal detected in time 0 stripped cells (*i.e.* no internalization) was averaged and subtracted from the other wells. The remaining signal detected after stripping treatment in the 60-min, 37 °C incubation wells was then normalized to the total surface staining in the unstripped wells and expressed as a percentage of total BK channel surface expression without stripping.

### Acyl-RAC of BK channel S-acylation

Acyl-RAC experiments were based on the protocol as previously described (15, 29). Transfected cells were lysed 48 h post-transfection in blocking buffer (100 mm HEPES, 1 mm EDTA, and 2.5% SDS, pH 7.5), disrupting cells using a 21-gauge needle and syringe for 20 strokes. The lysates were treated with 0.1% methanethiosulfonate and incubated for 4 h at 40 °C with shaking. The proteins were precipitated in acetone and stored at −20 °C overnight before washing five times in 70% acetone to remove the methanethiosulfonate and allow protein resuspension by dissolving precipitate in 300 μl of binding buffer (100 mm HEPES, 1.0 mm EDTA, 1% SDS, pH 7.5). After removal of 20 μl for input analysis, the remaining blocked proteins were divided into two tubes and treated with either 0.3 m hydroxylamine (NH_2_OH; Scientific Laboratory Supplies) or 0.3 m NaCl, pH 7.5. Thiopropyl–Sepharose beads were rehydrated in a 1:1 slurry in binding buffer, and 50 μl of beads was added to each tube, incubating for 2.5 h at room temperature. After bead capture, the samples were centrifuged at 13,000 × *g* for 1 min and washed five times in binding buffer. The proteins were then eluted in 50 μl of 2× SDS-LB heated to 60 °C for 10 min. Proteins were analyzed by 10% SDS-PAGE, loading 10 μl of input sample and 20 μl of eluted proteins, and probed by Western blotting for anti-FLAG. For quantitation, signals from the corresponding +NH_2_OH lane were first normalized to the input signal for that assay. The data for the effect of the respective thioesterase was then expressed as a percentage of *S*-acylation of the corresponding ZERO or STREX-C53A/C54A/C56A channel expressed alone.

### Membrane potential assay

Membrane potential assays were carried out as detailed previously for analysis of BK channel activity in HEK293 cells (22, 28). Transfected HEK293 cells were plated into a 96-well poly-d-lysine–coated, black-walled, clear-bottomed plate (Greiner) and assayed 48 h post-transfection. The cells were incubated with FLIPR® membrane potential blue dye (Molecular Devices, Sunnyvale, CA, USA) containing 2 mm CaCl_2_, for 30 min at room temperature. Assays were performed using the Flexstation R II system (Molecular Devices) at 22 °C by the addition of the calcium ionophore ionomycin (0.83 μm final concentration) after 16s. Fluorescent measurements as relative fluorescence units (RFU) were determined over a period of 180 s. In this assay an increase in BK channel activity results in a hyperpolarization that is detected as a decrease in fluorescence and is fully blocked by the specific 1 µm of the BK channel inhibitor paxilline (22). The assay is optimized to drive BK channel activation by elevation of intracellular free calcium that peaks approximately 40 s following ionomycin addition before declining (22). Because *S*-acylated STREX channels show increased apparent calcium sensitivity, without a significant change in voltage sensitivity compared with deacylated STREX-C645A/C646A channels (17, 18, 19, 22), the peak calcium induced hyperpolarizing response at ∼50 s represents the most sensitive component of the assay to discriminate functional *S*-acylation of STREX channels. To compare between conditions, the RFU for each well was first normalized to its basal (pre-ionomycin stimulus) RFU over the first 16 s or recording as shown in Fig. 5*A*. To allow quantification between experiments, as in Fig. 5*B*, the peak hyperpolarizing response in STREX expressing cells in each experiment was determined and subtracted from the corresponding HEK293 control, and the peak response was then expressed as a fraction of the peak hyperpolarization in cells expressing STREX channels alone.

### Statistics

The data are expressed as means ± S.D., *n* = number of independent experiments. Statistical analysis was performed, as appropriate, by one-way analysis of variance with Sidak post hoc multiple comparison tests for multiple comparisons of parametric data and using Kruskal**–**Wallis test with Dunn's post hoc test for nonparametric data (GraphPad Prism 8). Significant differences between groups were defined at *p* < 0.05 (*) and *p* < 0.01 (**).

## Data availability

All data are presented in the article or are available from the corresponding author (Michael J Shipston, University of Edinburgh, mike.shipston@ed.ac.uk) on request.

## Author Profile

Heather McClafferty
