# Supplementary material for: Site-specific deacylation by ABHD17a controls BK channel splice variant activity
Source: J Biol Chem. 2021 Jan 13;295(49):16487–96. doi: 10.1074/jbc.RA120.015349 (PMC7864050; doi:10.1074/jbc.RA120.015349)
Supplement: Supplementary file 1 — Heather McClafferty [file mmc1.pdf]

Heather McClafferty

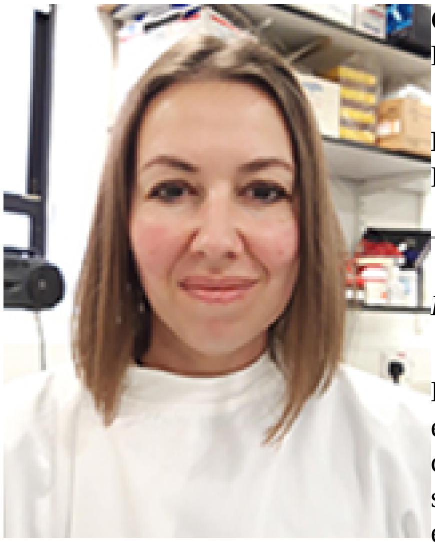

Current position: Research Associate, University of Edinburgh, Edinburgh, Scotland, United Kingdom

Education: B.Sc. in Medical Microbiology, 2000, University of Edinburgh, Edinburgh, Scotland, United Kingdom

How did you become interested in this topic?

My interest in this topic began when our group first discovered the dual effects of palmitoylation on the BK ion channel. While the pore of the channel that is common to all BK variants can be palmitoylated, a further site of S-acylation is encoded in the alternatively spliced stress-regulated exon (Strex). These two sites are modified by different DHHC enzymes and have different effects on the channel, thus giving a model protein in which we can investigate the effects of S-acylation and deacylation. The different mechanisms of action and regulation of this process may open up avenues for further understanding of the effects on the channel biology.

Can you describe an exciting moment you experienced while doing this research?

The most satisfying point in this research was seeing the data emerge from the acyl-RAC experiments. Reproducibility using this technique can be a technical challenge that makes the interpretation of subtle differences difficult; however, that was not the case in these experiments.

If you could go back in time and re-do this project, what advice would you give your past self?

If I were to give my future self some advice on this project, then I would advise myself to have more patience and wait for all the data before judging whether something was a success or not. In the lab, I would switch all the epitope tags required at the beginning of the experiments to avoid cloning throughout the project!

What do you hope to do next?

It would be interesting to elucidate the selectivity mechanism by which the enzymes act on the proteins, which may be the future direction of this work.

Where do you seek scientific inspiration?

For inspiration, I see my office and lab colleagues as a huge source, thanks especially to their informal day to day chat and discussions on life, science, and everything!

Read McClafferty's article on page 16487.
